# ER stress activates immunosuppressive network: implications for aging and Alzheimer’s disease

**DOI:** 10.1007/s00109-020-01904-z

**Published:** 2020-04-11

**Authors:** Antero Salminen, Kai Kaarniranta, Anu Kauppinen

**Affiliations:** 1 Department of Neurology, Institute of Clinical Medicine, University of Eastern Finland, P.O. Box 1627, FI-70211 Kuopio, Finland; 2 Department of Ophthalmology, Institute of Clinical Medicine, University of Eastern Finland, P.O. Box 1627, FI-70211 Kuopio, Finland; 3 Department of Ophthalmology, Kuopio University Hospital, P.O. Box 100, FI-70029 Kuopio, Finland; 4 School of Pharmacy, Faculty of Health Sciences, University of Eastern Finland, P.O. Box 1627, FI-70211 Kuopio, Finland

**Keywords:** Ageing, Immunometabolism, Immunosenescence, Immunosuppression, Inflammaging, Neurodegeneration

## Abstract

The endoplasmic reticulum (ER) contains stress sensors which recognize the accumulation of unfolded proteins within the lumen of ER, and subsequently these transducers stimulate the unfolded protein response (UPR). The ER sensors include the IRE1, PERK, and ATF6 transducers which activate the UPR in an attempt to restore the quality of protein folding and thus maintain cellular homeostasis. If there is excessive stress, UPR signaling generates alarmins, e.g., chemokines and cytokines, which activate not only tissue-resident immune cells but also recruit myeloid and lymphoid cells into the affected tissues. ER stress is a crucial inducer of inflammation in many pathological conditions. A chronic low-grade inflammation and cellular senescence have been associated with the aging process and many age-related diseases, such as Alzheimer’s disease. Currently, it is known that immune cells can exhibit great plasticity, i.e., they are able to display both pro-inflammatory and anti-inflammatory phenotypes in a context-dependent manner. The microenvironment encountered in chronic inflammatory conditions triggers a compensatory immunosuppression which defends tissues from excessive inflammation. Recent studies have revealed that chronic ER stress augments the suppressive phenotypes of immune cells, e.g., in tumors and other inflammatory disorders. The activation of immunosuppressive network, including myeloid-derived suppressor cells (MDSC) and regulatory T cells (Treg), has been involved in the aging process and Alzheimer’s disease. We will examine in detail whether the ER stress-related changes found in aging tissues and Alzheimer’s disease are associated with the activation of immunosuppressive network, as has been observed in tumors and many chronic inflammatory diseases.

## Introduction

**Fig. 1:**
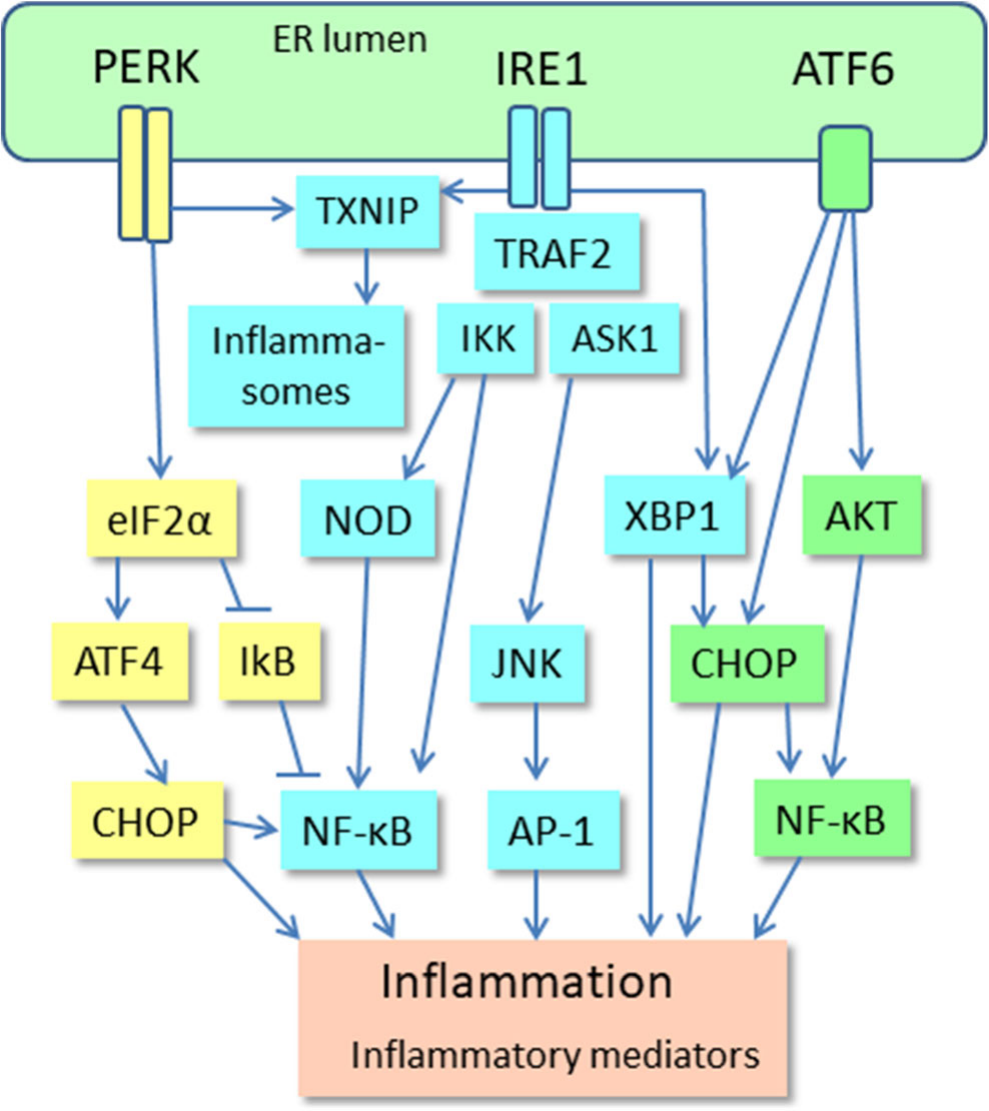
ER stress evokes inflammatory responses via the activation of three branches of ER stress sensors. PERK, IRE1, and ATF6 signaling pathways induce the expression of inflammatory mediators by activating AP-1, CHOP, and NF-κB transcription factors. IRE1 and PERK signaling can also trigger TXNIP signaling which activates inflammasomes. There are several review articles depicting the UPR pathways more thoroughly, both the basic pathways [1, 17, 26, 27, 31] and those related to inflammation and immunity [16, 81, 82]. Abbreviations: AKT, protein kinase B; AP-1, activator protein 1; ASK1, apoptosis signal-regulating kinase 1; ATF, activating transcription factor; CHOP, CCAAT-enhancer-binding protein homologous protein; eIF2α, eukaryotic initiation factor 2α; ER, endoplasmic reticulum; IκB, nuclear factor of kappa light polypeptide gene enhancer in B-cells inhibitor; IKK, IκB kinase; IRE1, inositol-requiring protein 1; JNK, c-Jun N-terminal kinase; NF-κB, nuclear factor-κB; NOD, nucleotide-binding oligomerization domain-containing protein; PERK, protein kinase RNA-like ER kinase; TRAF2, TNF receptor-associated factor 2; TXNIP, thioredoxin-interacting protein; XBP1, X-box binding protein 1

Host defence is based on diverse sensor systems which recognize different harmful stimuli and are able to induce specific adaptive responses in order to restore cellular homeostasis. Oxidative, proteotoxic, and metabolic stresses as well as hypoxia and impaired calcium balance are common inducers of endoplasmic reticulum (ER) stress. There are three branches of transmembrane ER sensors which detect disturbances in the quality of protein folding in the ER [1]. These are the following: (i) inositol-requiring protein 1 (IRE1), (ii) protein kinase RNA-like ER kinase (PERK), and (iii) activating transcription factor 6 (ATF6) which recognize the accumulation of unfolded and misfolded proteins in the lumen of ER (Fig. 1). Subsequently, these transducers trigger downstream cytoplasmic signaling cascades which induce the unfolded protein response (UPR). The function of UPR is to restore the quality of protein folding and protect cells against stress-induced injuries. If there is an overwhelming stress, UPR signaling can activate the processes that trigger apoptotic cell death. Moreover, it is crucial that UPR signaling is also able to generate alarmins, e.g., chemokines and cytokines, which recruit inflammatory cells into tissues suffering excessive stress. Chronic ER stress, defects in proteostasis, and inflammation are the typical features of many human diseases including metabolic diseases, inflammatory diseases, cancers, and several age-related diseases, such as atherosclerosis and neurodegenerative diseases [2, 3]. Currently, it seems that chronic ER stress is actually the major contributor to chronic inflammation rather than simply a consequence of inflammation.

Recently, it has been observed that both myeloid and lymphoid cells are able to exhibit extensive plasticity; for instance many immune cells display both the pro-inflammatory and anti-inflammatory phenotypes in a context-dependent manner, e.g., after infiltration of these cells into stressed tissues. The immune suppressive phenotypes are commonly called regulatory subtypes since they can inhibit the functions of immune effector cells [4, 5]. The best characterized immunosuppressive subtypes of myeloid cells include immature myeloid-derived suppressor cells (MDSC) as well as regulatory macrophages (Mreg/M2c) and dendritic cells (DCreg/tolDC). Correspondingly, regulatory T (Treg) and B (Breg) cells are immunosuppressive lymphoid cells. Inflammatory mediators are potent enhancers of immunosuppressive phenotypes, and chronic inflammation is generally associated with a compensatory immunosuppressive state. Recent studies have demonstrated that ER stress has a key role in the immunosuppression induced by the inflamed tumor microenvironment [6–8]. In fact, ER stress can be transmitted from the host tissue into recruited immune cells in inflamed tissues [8–10]. There is convincing evidence that ER stress induces the polarization of immune cells toward immunosuppressive phenotypes [8, 11]. In pathological conditions, it seems that the ER stress of host tissue can recruit immune cells and provoke inflammation in the acute phase, whereas in chronic inflammatory states, the ER stress activates immunosuppression to try to dampen excessive inflammation. Immunosuppression has many detrimental effects in persistent inflammatory disorders leading to immune deficiencies, cellular senescence, and tissue degeneration.

A chronic low-grade inflammation and impaired proteostasis are two hallmarks of the aging process and Alzheimer’s disease (AD). ER stress is closely associated with both aging and AD pathology (see below). For instance, ER stress is connected to the generation of cellular senescence [12–14]. The number of senescent cells progressively increases with aging. It is also known that ER stress is evident in neurons of human AD patients [15]. There is substantial evidence that the immunosuppressive network has become activated in the aging process and AD pathology (see below). Given that both the aging process and AD are associated with chronic inflammation and impaired proteostasis, it seems likely that ER stress induces immunosuppression which disturbs the maintenance of tissue homeostasis not only during aging but also in AD pathology.

### ER stress stimulates UPR signaling

The UPR signaling pathways can restore cellular homeostasis by inducing either adaptive gene expression or provoking inflammation as an alarming response to cell damage [1, 16]. The primary function of UPR is (i) to halt protein synthesis, (ii) to induce the degradation of misfolded proteins, (iii) to increase the folding capacity by stimulating the expression of ER chaperones, and (iv) to prepare immune cells for harmful conditions and imminent cell injuries. Moreover, UPR signaling can function in the cooperation with autophagy, proteasomes, and mitochondria [17–19]. For instance, ER stress can increase the autophagic degradation of cytosolic proteins or induce the selective, receptor-mediated ER-phagy [20, 21]. On the other hand, impaired autophagic degradation can trigger ER stress. Ghosh et al. [22] demonstrated that impaired autophagy in mouse adipose tissue from old animals was associated with increased ER stress and inflammation. The accumulation of misfolded proteins in the ER stimulates protein degradation via the ER-associated degradation (ERAD) pathway [17]. In the ERAD, misfolded proteins are ubiquitinated and subsequently transferred into the cytosol prior to proteasomal degradation. ER stress and UPR signaling also affect the crosstalk between ER and mitochondria at the mitochondria-associated membranes (MAM) where ER and mitochondria are physically in a close proximity [23]. This juxtaposition enhances calcium uptake into mitochondria, improves mitochondrial bioenergetic and redox balances, but it can also provoke apoptosis if there is excessive ER stress. There are many studies indicating that the disturbances in the MAM during ER stress might exert a significant role in the aging process and age-related diseases, such as Alzheimer’s disease [24, 25].

The signaling mechanisms of UPR have been reviewed in detail elsewhere [1, 26]. Briefly, the dimerization of IRE1α transducers by unfolded proteins stimulates their protein kinase domains in the cytoplasm, inducing the binding of TRAF2 protein to the IRE1α complex. Subsequently, TRAF2 can activate the IKK and ASK1 protein kinases which trigger the activation of the NF-κB signaling and the JNK pathway, respectively [1]. The IRE1-TRAF2-IKK signaling stimulates the expression of NF-κB-driven inflammatory genes which consequently activate the immunosuppressive cells in conditions of chronic inflammation (Fig. 1). The IRE1α protein also possesses endoribonuclease activity, cleaving mRNA molecules. The primary target of the regulated IRE1-dependent decay (RIDD) activity is the mRNA of X-box binding protein-1 (XBP1) cleaving off one intron, thus activating the translation of XBP1 mRNA. IRE1α can also split and degenerate distinct other mRNAs through the RIDD mechanism [27]. XBP1 is an important transcription factor which has many cell survival targets, but it can also evoke harmful effects. For instance, although it increases the expression of ER chaperones and ERAD components, it also exerts double-edged effects in glucose and lipid metabolism [28]. Interestingly, Martinez et al. [29] revealed that XBP1 improved mouse learning and memory processes by regulating memory-related genes, e.g., it increased the expression of BDNF. Recently, it has been revealed that the XBP1, an evolutionarily conserved protein, also has functions in cellular differentiation and cell cycle regulation which are not linked to ER stress [30].

The PERK transducer pathway is another signaling system which stimulates the UPR in ER stress [1, 31]. The cytoplasmic protein kinase domain of PERK protein phosphorylates eukaryotic translation initiation factor-2α (eIF2α) which inhibits protein synthesis and thus alleviates the pressure of unfolded proteins in the ER (Fig. 1). However, the phosphorylation of eIF2 kinase can enhance the translation of some stress-related mRNAs, e.g., activating transcription factor 4 (ATF4). Subsequently, ATF4 stimulates the expression of CHOP which in cooperation with ATF4 induces the expression of several autophagy genes [32]. The eIF2α kinase can also stimulate NF-κB signaling [33], the major inducer of inflammatory genes. There are observations indicating that the activation of PERK-eIF2α signaling has an essential role in memory deficits and Alzheimer’s pathogenesis, e.g., eIF2α activity elevates the expression of BACE1 and the production of β-amyloid [34]. The activation of PERK kinase also increases cell survival by activating NFE2L2 transcription factor which induces the expression of many cytoprotective genes [35]. In general, PERK signaling has beneficial effects to combat acute insults but its effects are detrimental if conditions become chronic, e.g., in mitochondrial quality control and metabolic responses.

The third branch of the UPR signaling pathways includes the ATF6 pathway and some other tissue-specific members of the bZIP family, e.g., CREBH found in liver and OASIS present in astrocytes [36]. ER stress triggers the translocation of ATF6 protein from the ER to the Golgi complex where its cytoplasmic domain becomes cleaved off by site 2 protease (S2P). The mature form of ATF6 is transferred into the nucleus where it acts as a transcription factor inducing not only stress-related genes, e.g., ER chaperones, ERAD components, autophagy genes, and CHOP factor but also the ER stress-unrelated developmental genes [36, 37]. ATF6 factor exists in two opposing isoforms, i.e., ATF6α is the transcriptional activator whereas ATF6β is the inhibitor. Interestingly, there is a close cross talk between different UPR signaling pathways. For instance, the PERK-ATF4 signaling increased the expression of ATF6 and enhanced its trafficking to the Golgi complex for the maturation process [38]. Correspondingly, ATF6 induced the expression of XBP1 mRNA which enhanced the signaling of IRE1 pathway [39].

### ER stress is associated with the aging process and Alzheimer’s disease

### ER stress in the aging process

The aging process is associated with a number of conditions known to induce ER stress, e.g., deficiencies in autophagic and mitochondrial functions, disturbances in Ca^2+^ homeostasis, increased oxidative stress, and disorders in proteostasis and energy metabolism [40]. However, studies on the expression levels of UPR proteins, both transcription factors and ER chaperones, have revealed contradictory results, as reviewed in detail by Estebanez et al. [41]. This is not a surprise since the aging process is a progressive state in tissues with both acute and adaptive changes induced by a variety of endogenous stresses and alterations in the microenvironment, e.g., metabolic, hormonal, and inflammatory changes. The UPR signaling controls protein quality and maintains proteostasis, i.e., it downregulates protein synthesis through the PERK-mediated phosphorylation of eIF2α and thus it prevents the accumulation of unfolded proteins in stressed cells. The decline of protein synthesis is the most characteristic hallmark of the aging process [40, 42]. However, PERK is not the only pathway which inhibits eIF2α kinase but other stress kinases of integrated stress response (ISR) can inhibit protein synthesis [43]. The UPR transducers can recognize the intensity of ER stress and thus induce dose-dependent responses, i.e., mild stress stimulates adaptation and cell survival responses, whereas a severe stress threatening cell death alerts the inflammatory system and triggers signaling leading to apoptosis. This indicates that ER stress can induce the dose-dependent hormetic responses [44]. There are observations that a mild ER stress, e.g., induced by dietary restriction, increased the activity of UPR and expanded lifespan [45]. It is also known that the preceding mild ER stress ameliorated the inflammation of endothelial cells induced by harmful insults, e.g., LPS treatment or excessive cytokine exposure [46, 47]. These observations indicate that the transducers of ER stress have a crucial role in the regulation of both health span and life span.

Several studies have revealed that the age-related tissue atrophy and degeneration are associated with a decline in the amounts of many UPR-related transducers and chaperones, whereas the expression of ER stress linked CHOP was significantly increased with aging, e.g., in mouse cerebral cortex [48] and adipose tissues [49] as well as in the skeletal muscles [50], liver [51], and retina [52] of rats. CHOP is not only associated with apoptosis, it performs many immune and metabolic responses as well as having effects on adipocyte and osteoblast differentiation [53]. For instance, the ER stress-induced expression of CHOP stimulated inflammation and consequently provoked insulin resistance in mouse adipose tissue [54]. In addition to the promotion of inflammation, Thevenot et al. [11] demonstrated that CHOP has a crucial role in the activation of the immunosuppressive properties of MDSCs in an inflamed tumor site, a microenvironment where there is sustained ER stress. For instance, CHOP stimulated NF-κB signaling in chronic inflammatory diseases, e.g., nonalcoholic steatohepatitis [55]. It seems likely that the age-related increase in the expression of CHOP is linked to inflammation rather than to apoptosis.

The aging process is associated with a progressively increasing accumulation of senescent cells in the tissues [40, 56]. Senescent cells commonly possess a flat morphology, and their cell cycle is irreversibly arrested. Senescent cells undergo a chromatin remodeling process, and they display the gene activation of the tumor suppressor network, e.g., p16INK4a and p53. Senescent cells also secrete inflammatory mediators, a phenomenon called senescence-associated secretory phenotype (SASP) [57]. It is known that diverse cellular stresses activate NF-κB signaling which triggers the inflammatory phenotype of senescent cells [58]. There is convincing evidence that ER stress is associated with cellular senescence although ER stress could be either the driver or a counteracting consequence of the senescent phenotype [12]. However, several experiments in vitro have revealed that the ER stress-induced UPR is rather the inducer of cellular senescence and not only an outcome. Druelle et al. [13] reported that ATF6α signaling controls many morphological aspects of the replicative senescence of human fibroblasts. The silencing of ATF6α signaling could partly reverse the ER expansion and SASP of senescent cells. Recently, Cormenier et al. [14] demonstrated that the activation of ATF6α maintained the senescent state of human fibroblasts by stimulating the COX2/PGE2 pathway. Most likely, ATF6α signaling triggered the prostaglandin pathway through the activation of NF-κB signaling. Oubaha et al. [59] reported that the IRE1 branch of UPR stimulated the SASP-induced pathological form of angiogenesis in mouse retinopathy. The IRE1α activation induced the senescent state in mouse retina by stimulating the RIDD pathway. The ribonuclease activity of IRE1 is an important mechanism in the control of protein synthesis, thus regulating cell homeostasis, e.g., in viral infections [27]. Currently, the selective elimination of senescent cells, called senolysis, is a promising therapeutic approach as it may alleviate the age-related diseases and thus extend life span [60].

### ER stress in Alzheimer’s disease

The pathogenesis of AD involves the gradual accumulation of β-amyloid plaques and neurofibrillary tangles in the human brain. The pathology of AD also involves clear evidence of inflammatory responses. The cause of impaired proteostasis in AD is still unknown although several hypotheses have been proposed. There is convincing evidence that ER stress is increased in human AD brains and might have a causative role in the pathogenesis of AD [61]. In their seminal study, Hoozemans et al. [62] demonstrated that the immunohistochemical staining of phosphorylated pPERK (activated) and UPR chaperone BiP/GRP78 were markedly increased in the neurons of temporal cortex and hippocampus in AD patients. In particular, the neurons of hippocampal CA1 and CA2 regions exhibited increased expression of pPERK and BiP/GRP78, whereas glial cells were not affected. Western blot assays revealed a clear correlation between the level of BiP/GRP78 and the amount of neurofibrillary tangles. In a later study, Hoozemans et al. [15] revealed that the UPR markers, i.e., pPERK, pIRE1α, and peIF2α, were clearly increased in the neurons containing the inclusions of granulovacuolar degeneration (GVD) in the hippocampus of AD patients. The most abundant pPERK staining was observed in those neurons which contained the diffuse localization of phospho-tau protein. They also observed that the increased staining of pPERK and pGSK-3β, a tau kinase, co-localized in same hippocampal neurons which indicates that UPR might stimulate tau phosphorylation. Subsequent studies have revealed that soluble tau protein can interact with several ER proteins, thus disturbing the function of ER and inducing UPR [63, 64]. Abisambra et al. [63] demonstrated that soluble tau protein interacted with the proteins of the ERAD complex. They also revealed that this interaction impaired the function of ERAD and thus triggered ER stress and stimulated the UPR. ER stress has been associated with the early phase of other tauopathies [65]. Fouillet et al. [66] reported that a preceding mild ER stress, i.e., preconditioning, was neuroprotective in the *Drosophila* and mouse models of Parkinson disease since it promoted neuronal autophagy. It seems that ER stress can exert protective effects in the early phase of AD.

AD is a progressive neurodegenerative disease leading to cognitive impairment and dementia in its later phases. There are studies indicating that β-amyloid peptides can induce ER stress [67]. Moreover, ER stress increases the activities of BACE1 and γ-secretase and thus enhances the processing of APP [68, 69]. Interestingly, Zhu et al. [69] demonstrated that the level of membralin (TMEM259), a key component of ERAD, was strongly downregulated in the brains of AD patients. Given that nicastrin is the substrate of membralin, they demonstrated that the knockdown of membralin in mouse hippocampus induced ER stress and increased the level of nicastrin, a component of the γ-secretase complex. The membralin deficiency increased γ-secretase activity and induced β-amyloid pathology as well as aggravating synaptic/memory deficits. It is not known whether the inhibition of ERAD by soluble tau (see above) might activate γ-secretase and induce AD pathology. There is abundant evidence emerging from animal experiments that the persistent activation of the PERK-eIF2α branch has a significant role in the memory and cognitive impairments encountered in AD and other tauopathies [34]. The activation of PERK phosphorylates eIF2α protein, thus inhibiting protein translation and consequently it disturbs the homeostasis of neurons. Duran-Aniotz et al. [70] observed that the level of activated IRE1 (pIRE1) correlated with the Braak stages of AD histopathology. Since it is known that IRE1 is activated in AD, these investigators generated a transgenic mouse model where the RNase domain of IRE1 was conditionally knocked out and then these mice were crossbreed with 5xFAD mice which exhibit extensive AD pathology. The ablation of IRE1 caused a significant reduction in the deposition of plaques and fibrillary tangles and an amelioration of the cognitive impairment as compared with the corresponding pathology present in the 5xFAD mice. The PERK and IRE1 branches are connected to the activation of NF-κB signaling, a potential neuronal trigger of AD pathology [71]. It seems that chronic inflammation might enhance AD pathogenesis through the activation of UPR signaling.

### ER stress is a potent inducer of inflammation

**Fig. 2:**
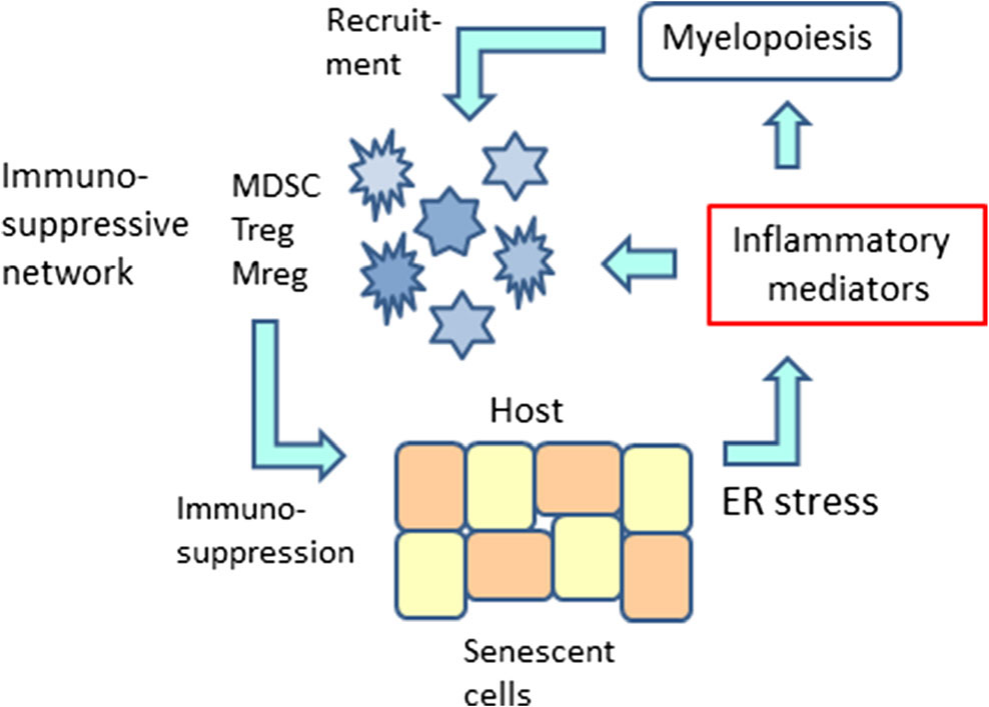
A schematic presentation on the induction of immunosuppression by ER stress. The ER stress of host tissues, containing, e.g., senescent cells, stimulates the secretion of inflammatory mediators which consequently provoke myelopoiesis in the bone marrow. Increased numbers of myeloid cells will be recruited into lymphoid and peripheral tissues. Inflammatory mediators secreted by host tissues trigger ER stress in infiltrated immune cells and concurrently enhance their immunosuppressive properties. A continual immunosuppression in inflamed tissues further impairs the homeostasis of tissues, e.g., by impairing proteostasis and enhancing cellular senescence. This ER stress-provoked vicious cycle degenerates tissues with aging and brains in Alzheimer’s disease. Abbreviations: MDSC, myeloid-derived suppressor cell; Mreg, regulatory macrophage; Treg, regulatory T cell.

The ER stress-induced UPR not only maintains cellular homeostasis but it can also defend tissues in chronic disturbances by inducing apoptotic cell death or secreting alarmins which provoke an inflammatory response. In acute injuries, inflammation restores tissue homeostasis but prolonged inflammatory conditions stimulate the immunosuppressive network in an attempt to prevent excessive inflammatory damage in tissues (see below). There is clear evidence that ER stress can elicit inflammatory responses in many age-related diseases, e.g., atherosclerosis [72], hepatic steatosis [73], and age-related macular degeneration [74]. It seems that the activation of inflammasomes is involved in the crosstalk between ER stress and inflammatory responses in some diseases [72, 73]. Given its role as an alarming mechanism, ER stress stimulates the secretion of chemokines and cytokines which subsequently recruit immune cells into tissues and activate their inflammatory function [75]. There are several reports indicating that ER stress could induce the expression and secretion of C-C motif chemokine ligand 2 (CCL2/MCP-1) in different cell types [76]. In addition, ER stress can stimulate the expression of several other chemokines and colony stimulating factors (CSF), e.g., those of CXC motif ligand 3 (CXCL3) in human endothelial cells [77] and granulocyte macrophage CSF (GM-CSF) in mouse adipocytes [78]. Kim et al. [79] demonstrated that the treatment with a CCR2 inhibitor reduced ER stress and lowered the inflammatory cytokine response in the steatotic liver of diabetic db/db mice. The treatment downregulated the infiltration of inflammatory cells into mouse liver, and it also improved insulin sensitivity. Interestingly, Kapoor et al. [80] reported that the protein induced by MCP-1, MCPIP, inhibited the activation of NF-κB signaling in mouse macrophages and thus mediated the M2 polarization which represents the anti-inflammatory phenotype of macrophages. In aged tissues, the senescent cells displaying the SASP phenotype could be a significant source of chemokine/cytokine secretion [57]. Chemoattractant proteins are important inflammatory mediators induced by ER stress since they enhance the expansion of immune cells and regulate their recruitment into the tissues undergoing stress (Fig. 2).

ER stress can induce inflammatory responses through all three branches of UPR via different signaling pathways [71, 81–83] (Fig. 1). However, the activation of NF-κB signaling is the crucial, cooperative pathway inducing pro-inflammatory responses. The activation of IRE1 kinase recruits TRAF2 adaptor protein to bind to its cytoplasmic domain. Subsequently, this complex interacts with several proteins which decide cellular fate in times of ER stress. For instance, IKK and TANK bind to the IRE1/TRAF2 complex and subsequently activate NF-κB signaling. Moreover, ASK1 kinase, a binding partner for TRAF2, exerts several context-dependent effects on inflammation, autophagy, and apoptosis [84]. ASK1 activates the expression of inflammatory genes through the JNK-AP-1 pathway (Fig. 1). Keestra-Gouder et al. [83] demonstrated that the two intracellular NOD receptors, NOD1 and NOD2, cooperate with the IRE1/TRAF2 complex to activate the NF-κB system in both macrophages and mouse tissues. These results indicated that NOD1/NOD2 signaling not only defends the organism against bacterial and viral infections but also protects cells from organelle stress. Kim et al. [85] reported that IRE1α activation also controlled the production of cytokines with the cooperation of XBP1 and GSK-3β in mouse macrophages. Interestingly, the splicing of XBP1 was associated with the expression of TNF-α, whereas the activation of GSK-3β increased the expression of IL-1β. It seems that the inflammatory responses induced by ER stress are specific and probably cell-type-dependent processes.

There is convincing evidence that ER stress activates inflammasomes via different mechanisms [86, 87] (Fig. 1). Inflammasomes are cytoplasmic multimeric signaling complexes which act as a host-defence system against pathogens and a self-defence mechanism to respond to cellular danger signals. Inflammasomes activate caspase-1 which cleaves the precursor forms of IL-1β and IL-18 into the mature, secreted pro-inflammatory cytokines. There are several types of inflammasomes containing different recognition sensors, e.g., NLRP1, NLRP3, and AIM2 [88]. It seems that ER stress can activate inflammasomes through different pathways in a context-dependent manner. Lerner et al. [86] demonstrated that the strong activation of IRE1α rapidly increased the level of thioredoxin-interacting protein (TXNIP) which subsequently activated NLRP3 inflammasomes in mouse pancreatic β cells (Fig. 1). They reported that IRE1α, a RIDDase enzyme, was able to split miR-17 which is a destabilizing factor of TXNIP mRNA. The activation of the IRE1α-TXNIP pathway promoted sterile inflammation and apoptosis in mouse β cells. The PERK signaling can also induce the TXNIP-mediated activation of NLRP3 inflammasomes in mouse β cells [89]. There are other mechanisms which can also trigger the ER stress-induced activation of NLRP3 inflammasomes. For instance, Robblee et al. [87] demonstrated that saturated fatty acids (SFA) induced ER stress in mouse and human macrophages. They reported that SFA activated IRE1α and subsequently stimulated NLRP3 inflammasomes through their binding to membrane phosphatidylcholines. These studies indicate that inflammasomes are an important target of UPR signaling.

The PERK pathways have many important functions in the generation of inflammatory responses (Fig. 1). For instance, Guthrie et al. [90] revealed that PERK signaling especially enhanced the ER stress-induced cytokine and chemokine production, whereas it had less effect on the UPR-dependent survival and adaptive responses in mouse astrocytes. Meares et al. [91] demonstrated that the ER stress-induced PERK signaling activated the JAK1-STAT3 pathway but it exerted a minor effect on the activation of NF-κB system in mouse astrocytes. Interestingly, STAT3 not only enhances pro-inflammatory reactions but it is the molecular hub of immunosuppressive responses in immune cells (see below). The robust activation of PERK and IRE1 by tunicamycin stimulated the expression of CHOP which induced pyroptosis, an inflammatory form of apoptosis, in mouse primary hepatocytes [92]. PERK stimulates CHOP signaling through the eIF2α-ATF4 pathway [93] (Fig. 1). CHOP can augment inflammatory responses by activating the NF-κB system, e.g., in human primary hepatocytes [55]. The PERK-eIF2α pathway can also activate NF-κB signaling by repressing the inhibitor of NF-κB (IκB) [94]. In addition, the activation of ATF6 branch can also stimulate the expression of inflammatory mediators via the AKT-NF-κB signaling [95] and the pathways between ATF6-XBP1-CHOP [1, 39] (Fig. 1). DeZwaan-McCabe et al. [96] revealed that the activation of CHOP in chronic inflammation promoted fibrosis and tumorigenesis in mouse liver. Conversely, the knockout of CHOP reduced the expression of inflammatory genes and prevented hepatic fibrosis and carcinogenesis. The activation of CHOP stimulates pro-inflammatory responses but it can also augment immunosuppressive properties [11], and thus in chronic inflammatory conditions, CHOP might control the balance between inflammation and immunosuppression.

### Chronic inflammation is a hallmark of aging process and Alzheimer’s disease

The aging process is associated with a chronic low-grade inflammation, a state called inflammaging [97, 98]. The signs of augmented inflammation are not only present in peripheral tissues but also in bone marrow and lymphoid organs [99, 100]. Recently, Benayoun et al. [101] reported that the transcriptional trajectories of many innate immune pathways were significantly induced with aging across different tissues in humans and rodents. For instance, interferon-α/γ responses, JAK/STAT3 and NF-κB signaling, and complement pathways were clearly upregulated with aging. Interestingly, they also revealed that specific epigenomic states were associated with distinct age-related transcriptional profiles. Currently, it seems that there are different sources for the activation of inflammatory responses with aging. Interestingly, certain types of cellular stresses, e.g., ER stress and mitochondrial disturbances, are associated with the activation of the immune system (see above). In addition, the number of cells displaying senescent phenotype increases in both bone marrow and peripheral tissues [56]. Senescent nonimmune cells secrete inflammatory mediators [57] which might be a significant source of chemokines and cytokines in tissues with aging. The aging process also affects the hematopoietic stem cells expanding the lineages of myelopoiesis and downregulating those of lymphopoiesis [102]. This myeloid-biased shift promotes the generation of myeloid cells in bone marrow and subsequently enhances their recruitment into aging tissues.

The pathogenesis of AD involves a clear activation of microglia and astrocytes on the affected brain regions [103]. However, it needs to be clarified whether inflammatory changes are a cause of AD pathology or a consequence of the accumulation of β-amyloid plaques. β-Amyloid peptides are potent inducers of innate immune responses via different pattern recognition receptors [104]. Nonetheless, randomized clinical trials with nonsteroidal anti-inflammatory drugs (NSAID) as well as the amyloid-based immunotherapies have been unable to prevent the pathogenesis of AD [105, 106]. Inflammasomes are activated in the AD pathology which might generate an inflammatory microenvironment in the AD brain [107, 108]. Given that ER stress can activate inflammasomes, it is not surprising that neuronal ER stress could be a trigger for inflammation and consequently induce AD pathology [71]. There are also studies indicating that certain infections, especially herpes simplex virus, could create conditions of chronic inflammation and consequently AD pathology [109]. However, experiments with transgenic AD mice have indicated that β-amyloid does seem to exert crucial role in AD pathogenesis. Currently, it is evident that chronic inflammation induces an antagonistic anti-inflammatory response, i.e., an immunosuppressive state, which while preventing excessive inflammation, may unfortunately enhance AD pathology (see below).

### ER stress-induced immunosuppression

### Inflamed microenvironment provokes immunosuppression

There is substantial evidence that inflammatory mediators have a crucial role in the activation of the immunosuppressive network in affected tissues. In particular, studies on tumorigenesis have revealed that inflammation is the driving force behind the recruitment and activation of immunosuppressive cells in tumor sites [110, 111]. MDSCs and Tregs are the most critical inducers of immunosuppression, i.e., they inhibit the functions of effector T cells and NK cells, and thus they allow tumor cells to escape immune surveillance. MDSCs are immature myeloid cells which are generated in myelopoiesis in bone marrow and subsequently secreted from bone marrow and recruited into extramedullary sites and inflamed tissues [110, 112]. There are two populations of MDSCs, i.e., monocytic MDSCs (M-MDSC) and polymorphonuclear MDSCs (PMN-MDSC), which have different immunosuppressive properties. Inflammatory mediators are potent inducers and activators of MDSCs [110]. Treg cells are the immunosuppressive subset of T cells which can be maturated in thymus or in peripheral lymphoid organs and also in inflamed tissues [113, 114]. Tregs are crucial regulators of innate and adaptive immunity, e.g., in self-tolerance. Tregs also has an important role in the maintenance of tissue homeostasis, e.g., in chronic inflammation [114].

Recent studies have revealed that ER stress has a significant role in the generation of immunosuppression in the tumor microenvironment [7, 8]. Interestingly, there is clear evidence that the ER stress present in cancer cells can be transmitted into the recruited myeloid cells in the tumor microenvironment [9, 115]. Mahadevan et al. [115] demonstrated that the UPR and the increased secretion capacity of inflammatory factors by tumor cells could be transmitted via a conditioned medium into bone marrow-derived dendritic cells (DC). These imprinted DCs expressed the markers of ER stress and secreted several pro-inflammatory cytokines, and moreover, they displayed an impaired antigen presentation and cross-priming of CD8^+^ T cells. The injection of these imprinted DCs into mice enhanced tumor growth. Several studies have revealed that it is not only tumor cells which can transmit ER stress into myeloid cells. For instance, Zhang et al. [10] demonstrated that the ER stress present in infected cardiomyocytes was able to transmit both the ER stress and pro-inflammatory properties into macrophages via soluble molecules but cell-cell contacts were not involved. In addition, ER stress can be transmitted between astrocytes, neurons, and microglia through soluble molecules in culture conditions [116]. It is known that ROS compounds and inflammatory mediators, e.g., IL-1β, TNF-α, and HMGB1, are able to induce ER stress in several nonimmune cells [117–119]. It seems that there is a positive feedback loop between ER stress and inflammation. Inflammatory mediators can maintain and augment ER stress in inflamed tissues. Santos and Ferreira [120] speculated that this kind of loop might aggravate AD pathology. Currently, the molecular mechanisms of ER stress transmission remain to be clarified although inflammatory mediators and exosomes (see below) might have a crucial role in transmission. For instance, Hosoi et al. [121] revealed that the ER stress-induced exosomes contained both spliced and unspliced XBP1 mRNAs. In conclusion, it seems that if there is ER stress in host tissues, this induces the secretion of pro-inflammatory factors and exosomes which not only recruit myeloid cells into inflamed tissues but are also able to potentiate the immunosuppressive properties of infiltrated myeloid and lymphoid cells (Fig. 2).

Acute and chronic inflammatory insults are associated with compensatory anti-inflammatory responses. Many detrimental conditions, e.g., traumatic injuries and pathogen-induced sepsis, induce a systemic inflammatory response syndrome (SIRS) which concurrently provokes a compensatory anti-inflammatory response syndrome (CARS) [122, 123]. The CARS state is linked with the activation of immunosuppressive cells, e.g., MDSCs, Tregs, and Mregs, which secrete anti-inflammatory cytokines, such as TGF-β, and IL-10 [124, 125]. The immunosuppression present in the SIRS/CARS states, e.g., in sepsis and many autoimmune diseases, can increase the risk for persistent infections and multiple organ failure. However, not only severe insults but all chronic inflammatory disorders can provoke an immunosuppressive condition which counteracts the pro-inflammatory responses, thus alleviating the destructive effects of persistent inflammation [4, 111, 126]. Many pathological conditions involving chronic inflammation display both pro-inflammatory and anti-inflammatory characteristics, i.e., they create a local SIRS/CARS state. For instance, an increased level of immunosuppressive cells has been observed in obesity-driven inflammation [127], psoriasis [128], and neuroimmune diseases [129]. These states also involve an ER stress although the dynamics between immune cells and host tissue still needs to be clarified.

### ER stress augments the immunosuppressive phenotype of immune cells

Infiltrated myeloid and lymphoid cells possess impressive plasticity which means that the microenvironment has an important role in the control of immune responses in tissues. The role of microenvironment in the activation of immunosuppressive network has been intensely studied in tumor biology. Tissue macrophages possess a remarkable functional plasticity in their capacity to adapt to alterations in tissue microenvironment [130, 131]. Moreover, circulating monocytes recruited into inflamed tissues can be differentiated into macrophages. Macrophages can be polarized between the pro-inflammatory M1 phenotype and the anti-inflammatory M2 state. However, there exists a continuum of properties rather than a dichotomy between M1 and M2 phenotypes. Additionally, the M2 phenotype includes M2a, M2b, M2c, and M2d subtypes which have specific functional properties [130]. Commonly, the M1 macrophages are activated in acute inflammatory conditions, whereas M2 subtypes are involved in the resolution of inflammation as well as tissue repair [131]. The number of immunosuppressive macrophages, especially regulatory M2c subtype, increases in delayed inflammatory conditions and they secrete anti-inflammatory cytokines, e.g., IL-10 and TGF-β. Many other immune cells, i.e., T and B cells as well as dendritic, natural killer, and natural killer T cells, also involve regulatory, immunosuppressive phenotypes [132]. It is known that inflammatory mediators, e.g., those secreted by the ER-stressed nonimmune cells, are the major inducers of immunosuppressive phenotypes in infiltrated myeloid cells [111, 112, 133]. For instance, MDSCs can be activated by the exposure to several cytokines, e.g., IL-1β, IL-6, IL-18, MIF and TNF-α, complements C3 and C52, as well as many alarmins, such as HMGB1, HSP70, PGE2, and S100A8/a9. Moreover, ER stress stimulates the release of extracellular vesicles and exosomes which contain, e.g., danger-associated molecular patterns (DAMP) and noncoding RNAs [134, 135]. Exosomes augment the expansion of MDSCs and Tregs and thus they are crucial enhancers of immune suppression in tumors [136]. In addition, tumor-derived exosomes can suppress the functions of effector immune cells, e.g. dendritic and NK cells. It seems that noncoding miRNAs are essential in the generation of immune suppression in tumors [135, 136]. Interestingly, exosomes might have an important role in Alzheimer’s pathology [137]. In the aging process, especially senescent cells possess an increased ability to secrete extracellular vesicles as a part of the SASP [138]. Given that exosomes contain a diverse set of molecules, it is still unknown how exosomes might promote tissue immunosuppression.

In the immune cells, ER stress contributes to many physiological functions, e.g., the development and differentiation of cells, although in a context-dependent manner, ER stress can activate immune responses [16]. Briefly, the intensity and prolongation of ER stress in infiltrated myeloid and lymphoid cells influence the immune responses, i.e., a severe ER stress promotes the immunogenic cell death (ICD), whereas chronic low-grade inflammation reinforces the tolerogenic and immunosuppressive properties of immune cells [8, 139]. Chronic inflammation stimulates the expansion of MDSCs, which are immature myeloid cells possessing a large set of immunosuppressive properties [112]. There are several studies indicating that ER stress in tumor cells increases the accumulation of MDSCs into tumor sites and enhances their immunosuppressive properties [11, 140]. Interestingly, Thevenot et al. [11] revealed that the expression of CHOP in MDSCs played a crucial role in the accumulation of these cells into mouse tumors and consequently CHOP was involved in the induction of their immunosuppressive activity. The expression of CHOP in MDSCs was mediated by ATF4 indicating that the PERK signaling pathway was activated. It is known that NFE2L2/NRF2 transcription factor is a direct substrate of PERK which stimulates the NFE2L2-induced cell survival mechanisms, e.g., antioxidant protection [35]. Beury et al. [141] demonstrated that NFE2L2 regulated the survival and function of mouse MDSCs, especially guarding MDSCs against oxidative stress in inflammatory microenvironments. Thevenot et al. [11] demonstrated that the activation of CHOP in MDSCs induced the stimulation of IL-6/STAT3 signaling and the expression of ARG1. It seems that the induction of CHOP in MDSCs is not linked to apoptosis but instead to the increase in immunosuppressive activity and the survival of MDSCs in inflamed tissues.

It is known that ER stress affects the polarization of immune cells, thus affecting their inflammatory properties. Franco et al. [142] revealed that the treatment of human Treg clones with thapsigargin, an ER stress activator, induced a strong increase in the expression of CHOP and IL-10. The induction of anti-inflammatory IL-10 was suppressed by salubrinal, an eIF2α-mediated inhibitor of ER stress, indicating that the PERK-eIF2α signaling is involved in the increase of immunosuppressive activity of Tregs. In contrast, Xu et al. [143] observed that the activation of the IRE1α-TRAF2 pathway through the deletion of Hrd1 protein impaired the expression of FOXP3 and also the immunosuppressive activity of mouse Tregs. Currently, the role of ER stress in the modulation of Treg activity is poorly understood. There is convincing evidence that ER stress can control the polarization of macrophages. Oh et al. [144] demonstrated that ER stress in human macrophages, induced by the exposure to either cholesterol or thapsigargin, promoted the shift from the pro-inflammatory M1 phenotype into the anti-inflammatory M2 subset. They also observed that the IRE1α-CHOP pathway was critical for M2 polarization and the subsequent increase in cholesterol uptake and the formation of foam cells. Furthermore, Suzuki et al. [54] reported that the macrophages isolated from the adipose tissue of CHOP knockout mice exhibit a significant increase in the level of the M2 subtype. It seems that the polarization of macrophages might be controlled in tissue specific manner as a response to the distinct pathology of host tissues. In this respect, it is interesting that in the inflammatory microenvironment of tumors, macrophages display the immunosuppressive M2-like phenotype, called the tumor-associated macrophages (TAM) [145]. Given the plasticity of macrophages and microglia, it is plausible that the specific macrophage/microglia subsets could appear in chronic inflammatory conditions, e.g., in aged tissues and Alzheimer’s disease.

The main function of dendritic cells (DCs) is to process antigens and subsequently present them on their cell surface to T and B lymphocytes. It is recognized that the ER stress present in DCs impairs both the processing of antigens and their presentation [115, 146]. Interestingly, Cubillos-Ruiz et al. [6] reported that the XBP1 signaling was robustly activated in the DCs located in the human and mouse ovarian tumors. They revealed that the products of lipid peroxidation generated by tumor cells induced ER stress in DCs involving the strong activation of IRE1-XBP1 signaling. Cubillos-Ruiz et al. [6] also demonstrated that the activation of XBP1 disrupted homeostasis within DCs, i.e., it triggered abnormal lipid accumulation, inhibited antigen presentation, and reduced T cell activation. These changes indicate that the stimulation of IRE1-XBP1 signaling switched the immunosuppressive phenotype of DCs and thus enhanced tumor progression. The ER stress-induced suppression of DCs has also been observed after a severe thermal injury in mice [147]. Zhu et al. [147] reported that a burn injury promoted the appearance of ER stress in splenic DCs which reduced the maturation of these cells and increased their apoptosis. The splenic DCs from injured mice also displayed a reduced capacity to enhance T cell proliferation and Th1 polarization. Treatment with salubrinal ameliorated the functional properties of splenic DCs after the thermal injury. It is known that this kind of injury provokes a systemic immunosuppressive response [148]. In conclusion, the ER stress present in inflamed host tissue enhances the recruitment of immune cells into tissues in attempts to terminate the inflammation but in chronic conditions, the pro-inflammatory phenotypes of immune cells will be switched into immunosuppressive subsets to prevent excessive tissue pathology.

### Activation of immunosuppressive network with aging and Alzheimer’s disease

The functional efficiency of human immune system declines with aging [149, 150]. The condition has been termed immunosenescence although it seems likely that immunosenescence is not the same phenomenon as the cellular senescence occurring in nonimmune cells. As long ago as the 1970s, the first studies appeared indicating that the defects in the immune system with aging were induced by an increased activity of immunosuppressive cells [151, 152]. There is some clinical evidence that the ageing process is associated with enhanced immunosuppression. For instance, (i) the risk for cancers is enhanced in elderly people, (ii) the efficiency of immunotherapy declines with aging, (iii) transplantation tolerance increases during the aging process, (iv) elderly people are vulnerable to infections, and (v) vaccination efficacy decreases during the aging process [153–155]. All these phenomena are associated with increased immunosuppression. Moreover, the survivors of cancer display a premature aging process [156] which may be caused by the tumor-induced systemic immunosuppression or treatments provided to combat the cancer which are known to provoke ER stress [157] and thus probably induce subsequent immunosuppression.

Currently, there is convincing evidence that the numbers of immunosuppressive MDSCs increases with aging in the blood of both humans [158, 159] and mice [160]. Several studies have also revealed an age-related increase in the accumulation of MDSCs into mouse bone marrow, spleen, and lymph nodes [160, 161]. Considering the impressive plasticity and differentiation capacity of immature MDSCs, it may be difficult to calculate their actual abundance in peripheral tissues. However, Ruhland et al. [162] observed that the number of MDSCs was augmented in the skin of elderly people. Correspondingly, the numbers of Tregs expand with aging in the blood and lymphoid tissues in both humans and mice [163–165]. It seems that the survival of Tregs becomes improved with aging, thus enhancing the expansion of Treg population. An age-related increase in the numbers of Tregs has also been observed in mouse visceral adipose tissue [166] and human skin [167]. Aging affects also the polarization of macrophages in a tissue-specific manner. The immunosuppressive M2 phenotype increases with aging in the bone marrow, spleen, lungs, and skeletal muscles, whereas the pro-inflammatory M1 phenotype augments in the liver, heart muscle, and adipose tissues [168, 169]. Pathological changes accompanying aging might increase the level of the pro-inflammatory M1 phenotype, e.g., in heart and adipose tissues. There are also significant age-related alterations in the phenotypes of dendritic cells and NK cells [170, 171] although it needs to be clarified whether they represent immunosuppressive subtypes. Since the immunosuppressive network functions in a cooperative manner, it seems that the increased presence of MDSCs and Tregs with aging promotes the polarization of other members to adopt the immunosuppressive phenotypes. We have recently reviewed the phenotypes of immune cells including in the immunosuppressive network and changes in their phenotypes during the aging process [132].

Chronic inflammation and the simultaneous deposition of β-amyloid plaques indicate that immunosuppression could be associated with AD pathology. Currently, there are observations both for and against that proposal; these discrepancies might be attributable to the difficulties in examining immune cells which exhibit such significant plasticity in brain pathology. Several studies have indicated that the function of microglia is impaired in both transgenic AD mice and AD patients [172]. Microglial cells are unable to phagocytose β-amyloid aggregates provoking the deposition of neuritic plaques. It is also known that the overexpression of pro-inflammatory cytokines or the injection of LPS mitigates the deposition of β-amyloid in transgenic AD mice [173, 174]. Moreover, the exposure of anti-inflammatory cytokines, e.g., TGF-β1 and IL-10, enhances the deposition of β-amyloid in the brain of transgenic mice [175, 176]. It seems that AD pathology is associated with increased immunosuppressive activity intended to protect neurons against detrimental inflammatory responses, but on the other hand, it prevents the cleansing of β-amyloid plaques.

Microglial cells are resident immune cells in the brain, but myeloid and lymphoid cells can infiltrate into the brain and affect the functions of microglial cells, especially in pathological conditions. Microglia, similarly to macrophages in other tissues, can be polarized between the pro-inflammatory M1 and anti-inflammatory M2 states [177]. Weekman et al. [178] demonstrated that the transition from the pro-inflammatory M1 state into the mixed phenotype, involving M2a and M2c markers, increased β-amyloid deposition in transgenic AD mice. In particular, the infiltrated immunosuppressive cells could trigger the M1/M2 shift of microglia through the secretion of anti-inflammatory cytokines, e.g., TGF-β and IL-10. Mild ER stress induced the M1/M2b polarization of microglia and alleviated the LPS-induced inflammation in rat brain [179]. There are observations that the number of Treg cells becomes increased in the blood of the patients with mild cognitive impairment (MCI) and AD [180]. Moreover, Di Benedetto et al. [181] demonstrated that the number of FoxP3-positive cells, a key marker of Tregs, was significantly elevated in the hippocampus of post-mortem AD samples as compared with that of age-matched controls. A similar phenomenon was observed in the brains of transgenic AD mice. Recently, Thome et al. [182] reported that the frequency and immunosuppressive activity of MDSCs in blood markedly increased in the early phases of AD but were reduced in severe AD dementia. Interestingly, Baruch et al. [183] demonstrated that the transient depletion of FoxP3-positive cells increased the clearance of β-amyloid aggregates and alleviated inflammatory responses in transgenic AD mice. In conclusion, it seems that AD pathogenesis involves the activation of immunosuppressive network which suppresses the function of microglia and provokes the deposition of amyloid plagues. We have recently examined in detail the role of MDSCs in the pathogenesis of AD [184].

### Chronic immunosuppression disturbs the homeostasis of tissues

Immunosuppressive cells possess cell-type-specific armament of inhibitory tools involving (i) the secretion of a diverse set of immunosuppressive cytokines, (ii) the expression of contact-dependent checkpoint proteins, e.g., PD-1/PD-L1 and CTLA-4 receptors, and (iii) the expression of amino acids catabolizing enzymes, such as arginase 1 (ARG1) and indoleamine 2,3-dioxygenase (IDO), which can inhibit protein synthesis and thus prevent the proliferation of immune cells [112, 185]. The immunosuppressive network is cooperative since suppressive cells control the activity and polarization of other members in the network. Immunosuppressive cells have a fundamental role in the suppression of detrimental innate and adaptive immune responses and in the maintenance of immune self-tolerance. Overall, the activation of immunosuppressive cells, such as MDSCs and Tregs, has beneficial effects since it results in the resolution of acute inflammatory responses and the suppression of excessive inflammatory disorders, e.g., sepsis, autoimmune diseases, and transplantation [110, 112, 186]. However, in chronic inflammatory conditions, e.g. tumors, obesity, chronic infections, and neurodegenerative diseases, the impaired capacity of effector immune cells exerts detrimental effects on the maintenance of tissue homeostasis. The age-related immunosenescence evokes very similar changes in the phenotypes of immune cells as can be induced by the MDSC-driven immunosuppression [187]. Currently, it needs to be clarified whether the immunosenescent phenotype of immune cells is generated by the activation of the immunosuppressive network. However, ER stress stimulates the secretion of the same chemokines which are known to recruit MDSCs into inflamed tissues and trigger immunosuppression [188].

Immunosuppressive cells secrete anti-inflammatory cytokines, e.g. TGF-β and IL-10, as well as reactive oxygen species (ROS). These factors are not only immunomodulators but they are also able to induce bystander effects in the cells of the host tissue. TGF-β receptors 1 and 2 (TGFBR1/2) are commonly expressed in human tissues (Human Protein Atlas). TGF-β cytokines trigger complex signaling pathways, mainly mediated via SMAD factors, which control several homeostatic functions, e.g. cell proliferation, differentiation, and senescence [189]. Fibrosis is probably the most common pathological change induced by TGF-β signaling in several tissues [190]. Interestingly, ER stress is involved in the pathogenesis of many fibrotic diseases [191]. In particular, the myocardium and lungs are affected by fibrosis associated with aging. In some tissues, the ER stress-induced fibrosis also involves the appearance of myofibroblasts, i.e., the cells which secrete excessive amounts of extracellular matrix components, producing atheromatous plaques in blood vessels. TGF-β is a potent inducer of the differentiation of fibroblasts into myofibroblasts [192]. It has been known for a long time that TGF-β stimulates the expression of collagen and fibronectin by fibroblasts and thus it remodels the extracellular matrix [193]. In mouse skeletal muscles, TGF-β provoked the appearance of endomysial fibrosis, collagen accumulation, and myofiber atrophy [194], common characteristics of sarcopenia.

In addition to fibrosis, TGF-β signaling is a potent inducer of cellular senescence [195]. The responses might be both cell type-specific and context dependent. Senturk et al. [196] reported that the administration of TGF-β induced the p15Ink4b and ROS-dependent senescent arrest in hepatocellular carcinoma cells and also inhibited tumor growth in mouse model. This effect is not only present in tumor cells since TGF-β exposure can also trigger cellular senescence in human diploid fibroblasts and mouse keratinocytes [197, 198]. Frippiat et al. [197] demonstrated that the H_2_O_2_-induced oxidative stress stimulated the expression and secretion of TGF-β1 in human fibroblasts. The H_2_O_2_-induced cellular senescence was inhibited in a TGFBR2-dependent manner. It is known that ROS compounds are important mediators of the MDSC-induced immunosuppression [199]. Several studies have revealed that the activation of MDSCs induced a robust increase in the secretion of ROS compounds through the activation of NADPH oxidase (NOX2) [199]. Interestingly, MDSCs themselves are resistant to oxidative stress since they have a significant level of NFE2L2-induced antioxidants [141]. It seems that immunosuppressive cells not only can arrest the proliferation of immune cells but TGF-β and other immune modulators can also induce harmful bystander effects in host tissues.

Amino acid catabolism is an important mechanism in the control of immunosuppression [200, 201]. Given that many immune cells are auxotroph for certain amino acids, especially arginine and tryptophan, this means that the expression of the catabolic enzymes of these amino acids, i.e., ARG1 and IDO, by immunosuppressive cells restricts the function of auxotrophic immune cells in inflamed tissues. The amino acid shortage induced by immunosuppressive cells inhibits the function of many immune cells, e.g., T lymphocytes, but at the same time, it affects the cells of the host tissue although many neighboring cells are able to synthesize arginine but not tryptophan for their own purposes. This amino acid deficiency stimulates the GCN2 kinase which subsequently restricts protein translation via the phosphorylation of eIF-2α [202]. In addition, the activation of ARG1 and IDO generates active metabolites which can affect the functions of inflamed host tissues. For instance, arginine is a shared substrate for ARG1 and nitric oxide synthase (NOS) and thus the increase in ARG1 activity reduces the generation of NO by NOS [203]. The activation of ARG1 also produces urea and augments polyamine synthesis. It has been observed that the ARG1 activation could promote the shift of macrophages to adopt an anti-inflammatory M2 phenotype, whereas the activation of NOS stimulated the pro-inflammatory M1 functions [203]. The tryptophan catabolism by the IDO of immune suppressive cells stimulates the kynurenine pathway and the production of many active metabolites, e.g., quinolinic and kynurenic acids, which are involved in the pathogenesis of several diseases including many neurodegenerative diseases [204]. There are disturbances in arginine metabolism in the aging process and age-related diseases [205] as well as in Alzheimer’s disease [206]. Correspondingly, it seems that the kynurenine pathway has an essential role in many age-related diseases [207] and Alzheimer’s disease [208]. In conclusion, the ER stress-induced inflammation, both during the aging process and many accompanying chronic diseases, generates an immunosuppressive state which not only prevents excessive inflammation but also insidiously degenerate host tissues as a consequence of the function of immune suppressive armament.

## Conclusions

ER stress induces hormetic responses which are subject to the intensity of stress, i.e., mild ER stress augments stress tolerance, whereas excessive stress can trigger apoptosis or evoke necrotic injuries. ER stress is able to generate alarming factors which alert the immune system for the probable cell injuries. The immune response involves the activation of the tissue-resident immune cells and the recruitment of myeloid and lymphoid cells into affected tissues. This means that the ER stress of the nonimmune cells can alert the immune system which subsequently will launch an acute inflammatory response in tissues. However, inflammatory responses, especially in chronic conditions, trigger a compensatory immunosuppression in an attempt to prevent more severe tissue damage. There is convincing evidence that the ER stress of host tissues can trigger ER stress in immune cells which affects the properties of immune cells, shifting them toward the anti-inflammatory phenotype. This promotes the resolution of inflammation in acute states. In chronic conditions, where the insult cannot be removed, e.g., in the aging process or Alzheimer’s disease, this state of immunosuppression has a crucial role in the prevention of detrimental inflammatory injuries. However, a persistent ER stress can trigger cellular senescence in inflammaging tissues or aggravate pathological processes underlying Alzheimer’s disease. On the other hand, the mediators of immunosuppression secreted by immune suppressive cells, e.g., TGF-β, IL-10, and ROS, induce degenerative bystander effects in the affected tissues. This scenario indicates that ER stress has a crucial role in the control of the balance between inflammatory and immunosuppressive responses. This has been speculated to be present in many pathological processes but it also seems to occur in the inflammaging process and in Alzheimer’s disease.
